# Direct visualization of local activities of long DNA strands via image–time correlation

**DOI:** 10.1007/s00249-021-01570-0

**Published:** 2021-09-09

**Authors:** Kyongok Kang, Yue Ma, Koichiro Sadakane

**Affiliations:** 1 Biomacromolecular Systems and Processes, Institute of Biological Information Processing, IBI-4, Forschungszentrum Jülich, Jülich, Germany; 2 Faculty of Life and Medical Sciences, Doshisha University, Kyotanabe, 610-0394 Japan

**Keywords:** Bacteriophage long DNA strands, Thermal translocations, Condensed state of DNA globules, image–time correlation, Hydrophobic antagonistic salt, Elasticity of motile DNA strands

## Abstract

Bacteriophages with long DNA genomes are of interest due to their diverse mutations dependent on environmental factors. By lowering the ionic strength of a hydrophobic (PPh4Cl) antagonistic salt (at 1 mM), single long T4 DNA strand fluctuations were clearly observed, while condensed states of T4 DNA globules were formed above 5–10 mM salt. These long DNA strands were treated with fluorescently labeled probes, for which photo bleaching is often unavoidable over a short time of measurement. In addition, long (few tens of $\upmu m$) length scales are required to have larger fields of view for better sampling, with shorter temporal resolutions. Thus, an optimization between length and time is crucial to obtain useful information. To facilitate the challenge of detecting large biomacromolecules, we here introduce an effective method of live image data analysis for direct visualization and quantification of local thermal fluctuations. The motions of various conformations for the motile long DNA strands were examined for the single- and multi-T4 DNA strands. We find that the unique correlation functions exhibit a relatively high-frequency oscillatory behavior superimposed on the overall slower decay of the correlation function with a splitting of amplitudes deriving from local activities of the long DNA strands. This work shows not only the usefulness of an image–time correlation for analyzing large biomacromolecules, but also provides insight into the effects of a hydrophobic antagonistic salt on active T4 bacteriophage long DNA strands, including thermal translocations in their electrostatic interactions.

**Supplementary Information:**

The online version contains supplementary material available at 10.1007/s00249-021-01570-0.

## Introduction

The bacteriophage T4 capsid carrying its DNA genome consists of several functional regions including a structural RNA. The T4 tail has a contractile rigid tube and a terminating multiprotein baseplate, with phage DNA transferring from the capsid into a host cell through the tail tube via sheath contraction (Rossmann et al. 2004). The T4 DNA structure remains condensed after the structural RNA and protein components have been removed (Hamilton and Pettijohn 1976). T4 DNA ligase is an enzyme that is widely used in molecular biology to repair chromosomal DNA during mutagenesis (Wang et al. 2019). The distribution of covalent modifications in T4 DNA is inhibited by single-molecule DNA sequencing and enzymatic probing (Bryson et al. 2015). Details of the structural analysis of bacteriophage T4 DNA replication have been reported, consisting of 10 proteins known collectively as a replisome, which is responsible for the replication of the phage genome (Mueser et al. 2010). This is much more complex than for the *E. coli* chromosome. The bacteriophage T4 replisome can be subdivided into two components: the DNA replisome and the primosome (Nossal 1994). The DNA packaging mechanism of bacteriophage T4 is performed by a terminal motor docked to a procapsid portal via several specific sequence-determined contacts (Black 2015). The cryo-EM structure of the portal assembly of T4 is known in atomistic resolution (Sun et al. 2015). Interestingly, large force generations with high velocity are detected by force-clamp measurements in the motor proteins for DNA translocation dynamics via tensile length (in kbp) and the average velocity of 700 bp/s up to 2000 bp/s (Fuller et al. 2007). Similar findings relating to the high speed of the DNA packaging motor of bacteriophage T4 have been observed via optical tweezers (Lin et al. 2017). Maintaining such a high speed of the molecular motor is crucial to functions being sustained. Recently, two hydrophobic amino acid residues in the catalytic space of gp17-ATPase were found to render motor speed much less than the wild-type motors (Lin et al. 2017). Reversible condensation and decondensation of DNA strands often exhibits complexity in the transduction of ATP hydrolysis energy into mechanical motions of DNA (Rao and Black 2013). As a potential condensation mechanism, the precipitation of highly charged polyelectrolyte solutions has been discussed in relation to the effective short-range electrostatic attraction between monomers (De la Cruz et al. 1995). T4 DNA compaction was shown by varying cationic species between the coil and globule formations (Zinchenko and Yoshikawa 2005). Differences in linear- and branched-chain polyamines were reported for the T4 DNA folding transition, from an elongated coil to a compact globule state that was demonstrated by the steric interaction (via Monte Carlo simulation) between negatively charged dsDNA and cationic polyamines (Kashiwagi et al. 2018). Significant temperature-dependent structural changes of T4 DNA were observed for the linear- and branched-chain polyamine via AFM images (Nishio et al. 2018). In addition, a single giant DNA strand was coated with gold nanoparticles, following Ref. (Carnerero et al. 2018), which allowed a probing of its dynamics by means of fluorescence microscopy, TEM, and CD spectra. Since the T4 DNA strands studied in this paper are quite large in size (a few tens of microns), their local motion corresponds to the application of image–time correlation spectroscopy (with a sampling time of 30 fps). Here, we have demonstrated the use of image–time correlation spectroscopy to analyze the real-time movie data of long DNA strands that are fluorescently labeled. The image–time correlation function then serves as a useful means of “quantifying” the fluctuating long DNA strands, over mesoscopic time scales (a few seconds), exploiting their elastic deformations directly from images of single- and multi-interacting strands in solution.

**Fig. 1:**
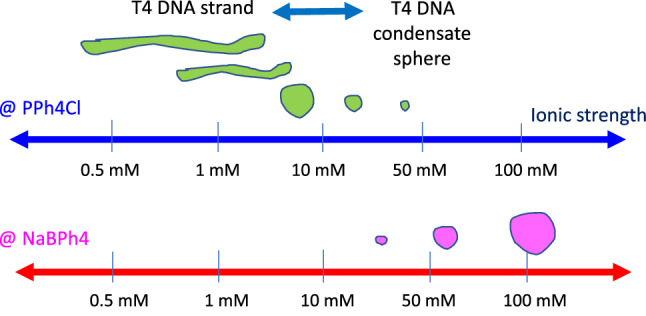
A simple schematic of the comparison between the positive (above) and the negative (below) moieties with hydrophobic antagonistic salt on the long T4 DNA strand and an increase of ionic strength. Interestingly, more stretched T4 DNA strands are observed, as an elongated coil or unfolded conformation, at a lower ionic strength of the cationic antagonistic salt (PPh4Cl) (below 1–5 mM) compared to the simpler feature of T4 DNA globules or compact conformations in the anionic antagonistic salt (NaBPh4)

**Fig. 2:**
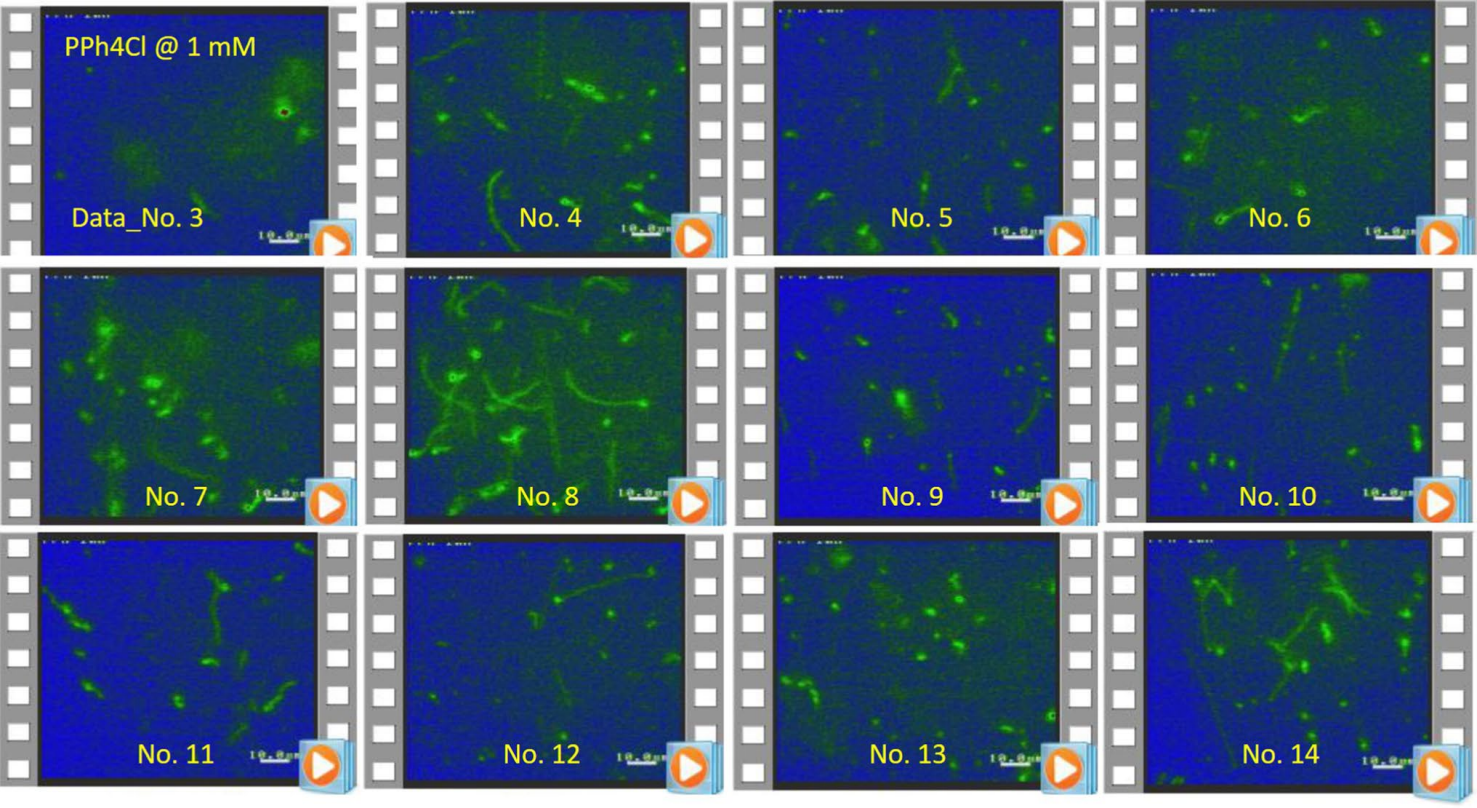
Various video microscopy data of T4 DNA strands, coexistent with T4 DNA globules, in the hydrophobic-cation antagonist salt (of 1 mM PPh4Cl salt) that were analyzed in this work via image–time correlation spectroscopy. Images are enhanced from a black/white TV camera using color-contrasting Fiji/ImageJ software. The scale bar in the bottom right is $10\;\upmu$m. The region of interest (ROI) is $360 \times 480$ pixels

Before we discuss the long T4 DNA strand fluctuations caused by hydrophobic-cation (PPh4Cl) salt, a comparison of positive and negative charged moieties of a hydrophobic antagonistic salt on the T4 DNA mixture was first tested. Figure 1 shows a simple illustration; the opposite trends of T4 DNA condensates are observed with an increase of the ionic strength of two different combinations of antagonistic salts: NaBPh4 is composed of Na+ (hydrophilic) and BPh4- (hydrophobic), while PPh4Cl is composed of PPh4+ (hydrophobic) and Cl- (hydrophilic). T4 DNA globules are clearly observed in the hydrophobic-anion antagonistic salt (NaBPh4). In contrast, stretched T4 DNA strands are observed—coexisting with T4 DNA globules—for the lower ionic strength (below 1–5 mM) of hydrophobic–cationic antagonistic salt (PPh4Cl). In this study, various optical morphologies of T4 DNA strands are presented at a low ionic strength of 1 mM PPh4Cl salt in Fig. 2, where all data movies are analyzed and discussed systematically. The results are shown to highlight the principles of image–time correlation spectroscopy for the data analysis of image–time correlations, which are set out in the Methods also, by emphasizing (i) the thermal fluctuation of various conformations, in mesoscopic time scales, for long T4 DNA strands, (ii) the elasticity of individual motile T4 DNA strands, and (iii) the electrostatic interactions of T4 DNA caused by varying the ionic strengths of a hydrophobic antagonistic salt (PPh4Cl). Finally, the discussion and experimental methods are given.

## Experimental methods

### Sample preparation

Bacteriophage T4 phage DNA (166 kbp, contour length $57\,\upmu$m) was purchased from Nippon Gene (Toyama, Japan). The persistence length of double-stranded DNA is in the order of 10 nm or 200 bp. Thus, long T4 DNA exhibits a contour length (or full length) that is 103 times larger than the persistence length, indicating its characteristic as a semiflexible polyelectrolyte. DNA samples were dissolved in a propanol–water solution for the final concentration of $30\,\upmu$M in nucleotide units. A fluorescent cyanine dye, YOYO-1 (quinolinium, 1, 1-[1, 3-propanediyl-bis [(dimethylimino)-3, 1- propanediyl] bis [4 [(3-methyl-2 (3H)-benzoxazolylidene)-methyl]]-tetraiodide) was purchased from Molecular Probes (Oregon, USA). YOYO-1 (final concentration: $1\, \upmu$M) was added to the DNA solution prior to observation. DNA molecules greater than tens of kilobase pairs behave as a semiflexible polymer, whereas short DNA molecules of less than several hundred base pairs are regarded as a stiff polymer. It is also rather difficult to handle long DNA molecules compared to short DNA fragments.

### The optical fluorescence video microscopy

Here, in our experiments, samples were illuminated with a 365 nm UV Mercury lamp (HBO 100, Carl Zeiss) for the fluorescence imaging of T4 DNA strands. The fluorescence images of DNA strands were observed using a Zeiss Axiovert inverted 135 TV microscope equipped with an oil-immersed 100x objective lens (Plan-Neofluar, 1.3 NA), and recorded on a DVD using a charged-coupled device (CCD) sensor (EBCCD) camera (C7190-43 series, Hamamatsu Photonics, Japan). The recorded videos were analyzed using VirtualDub (written by developer Avery Lee). All observations were made at around $25\, ^{\circ }$C. In this study, dynamic transitions of coil to globule are clearly demonstrated in a low ionic strength (1 mM hydrophobic–cationic antagonistic salt (PPh4Cl)) environment. Furthermore, as ionic strength increases, fewer condensate states of T4 DNA globules are observed due to DNA compaction, resulting in electrostatic interaction balances. In the mesoscopic time scale, thermal fluctuations of long motile T4 DNA strands are systematically analyzed by means of image–time correlation spectroscopy for Brownian motions.

### The image–time correlation spectroscopy

**Fig. 3:**
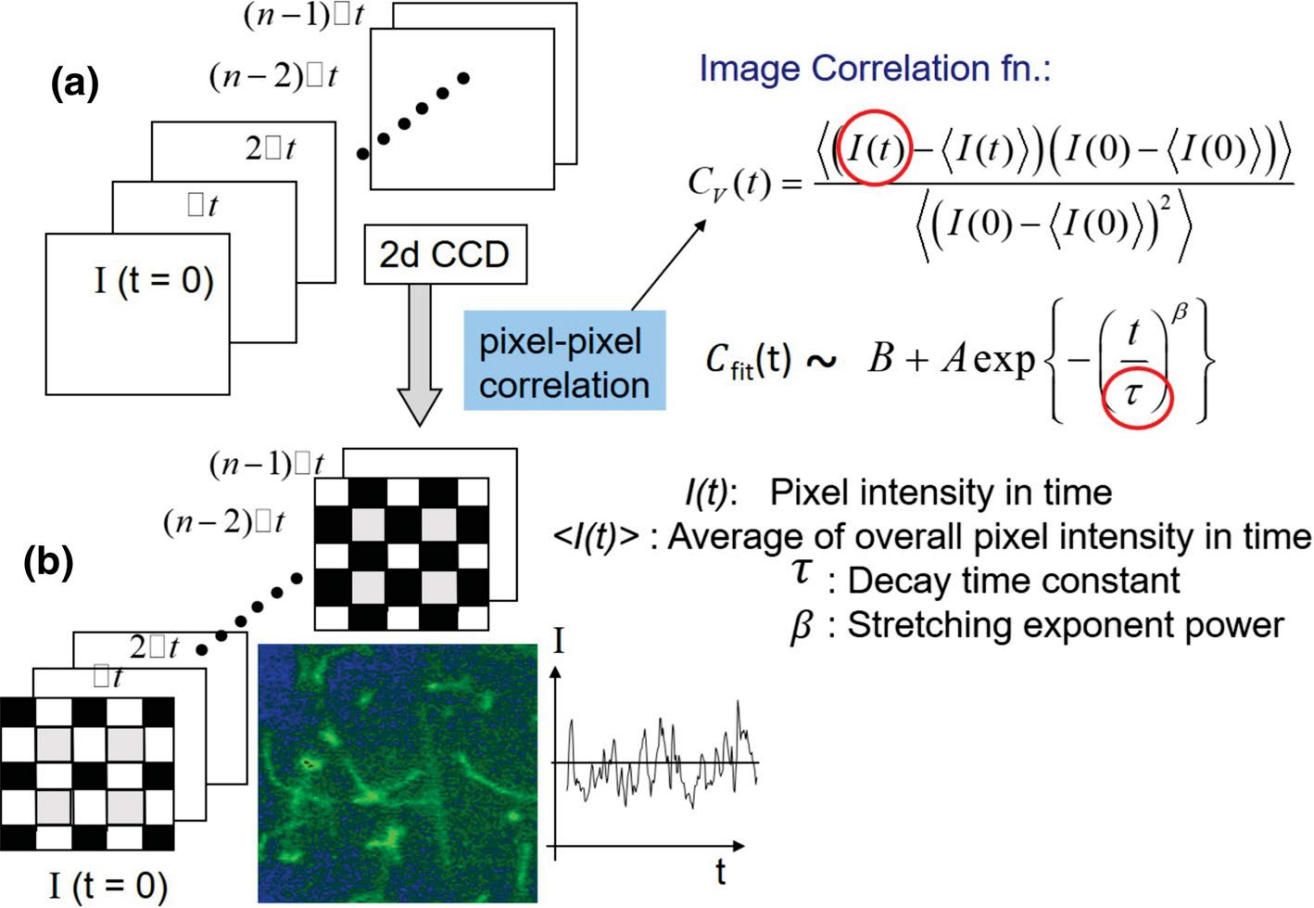
A simple schematic of the principles of image–time correlation spectroscopy: **a** Collection of time-lapse images and **b** reconstruction of image data in black/white binary ASCII formats to calculate the image–time correlation function from the pixel-pixel intensity auto-correlation. A 2D array of the square pixels are chosen as the typical region of interest (ROI) views

To quantify the morphological changes as probed by time-lapsed images, the correlation function for each pixel is calculated, and subsequently averaged over all pixels, before the calculation for an image–time correlation (Kang 2011). A simple schematic of the principles of image–time correlation spectroscopy is shown in Fig. 3, in which time-lapsed images are collected, followed by the reconstruction of image data in black/white 2D ASCII formats to calculate the image–time correlation function in the pixel-pixel intensity auto-correlation. The brief procedures for constructing the image–time correlation function were as follows: I(t) is the instantaneous transmitted intensity detected by a given pixel of the CCD camera. For the time traces recorded for all these pixels, the image–time correlation function $C_{V}(t)$ is defined as follows:

1 $$\begin{aligned} C_{V}(t) = \frac{<(I(t)-<I(t)>) (I(0)-<I(0)>)>}{<(I(0)-<I(0)>)^{2}>}\;, \end{aligned}$$

where the brackets $<\cdots>$ denote the averaging of the CCD camera pixels. Each single image in a time trace was used to construct an image correlation function that is variable for the region of interest in the square (e.g., $300 \times 300$) pixels. The measurement was done using a black and white camera, and its images were also used for the data analysis. Here, the presentation of movie data is enhanced by transformation into a 2D color map. In the analyses, intensity is read by each pixel as the high intensity due to fluorescence (as seen in green) against the non-fluorescent background (bluish). From the start of an experiment, the incident light intensity is chosen such that the recorded fluorescence intensity does not exceed the threshold where blooming (spill-over of the charge from pixel-sensor to neighboring pixel-sensors) occurs. In addition to this, for the calculation of the correlation function, the average over all pixels for each time is subtracted from each of the pixel-intensities, to correct for possible bleaching and variations of the incident intensity (Kang 2011). The above definition of an image–time correlation function is similar to a scattered auto-intensity correlation. More applications of image–time correlation spectroscopy to other systems can be found in Kang (2011, 2013, 2021), Kang et al. (2016, 2021).

Thus, in this work, the interest is in the direct visualization of local activities of conformational changes of individual long DNA strands, where we refrain from quantitative fits of correlation functions, for which the derivation of appropriate fitting functions depending on, for example, the elasticity of the chains would require a separate study. One of them is local amplitudes of fluctuating modulation that is obtained as a visible splitting in the data, resulting from repetitions of existing high and low values in the correlation, due to local motility of thermal Brownian motions for long T4 DNA strands. As can be clearly seen later, the most pronounced splitting occurs in the case of a single T4 DNA strand fluctuation, by the repeating motions between the fully stretched and partially contracted strands. The image–time correlation is obtained for the decays of the single and multiple T4 DNA strands at a low ionic strength of 1 mM hydrophobic-cation antagonistic salt (PPh4Cl) for various movie datasets (see Fig. 2), observed by fluorescent video microscopy experiments. As the ionic strength of the hydrophobic–cationic salt increases, condensed states of T4 DNA globules are formed, illustrated in the upper part of Fig. 1. In contrast, at a low ionic strength (of 1 mM), T4 DNA strands and condensed T4 DNA globules coexist. The aim of this paper is to demonstrate versatile T4 DNA conformation changes with antagonistic salt. In this present study, thermal translocations of T4 DNA strands (at an ionic strength of 1 mM PPh4Cl) are important for validating interactions, as well the elasticity of single T4 DNA strands relating to the motility captured in the image–time correlations.

## Results

### Thermal fluctuation of various conformations of T4 DNA strands at a low ionic strength of the hydrophobic salt

**Fig. 4:**
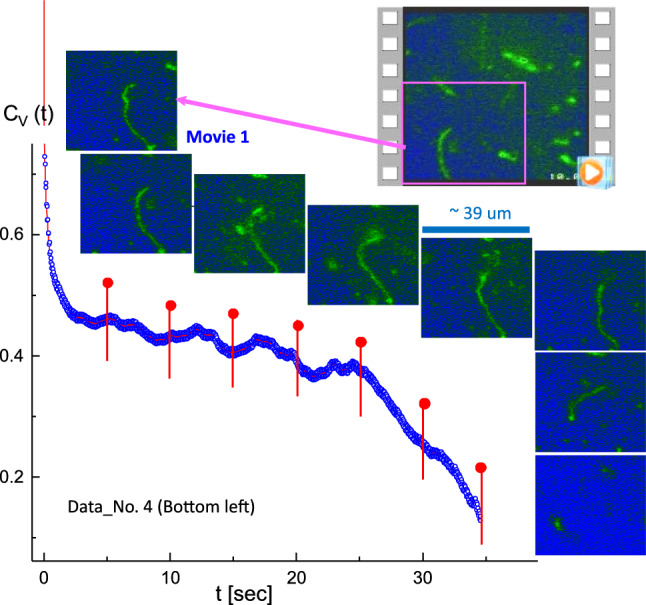
A stretched single T4 DNA strand is analyzed, with the corresponding time-lapsed images indicated as the red pins in the correlation function. This is a result of the image–time correlation in the bottom-left region of Data No. 4 (Fig. 2; Movie 1 in the supplementary data). The ROI is $250 \times 250$ pixels

**Fig. 5:**
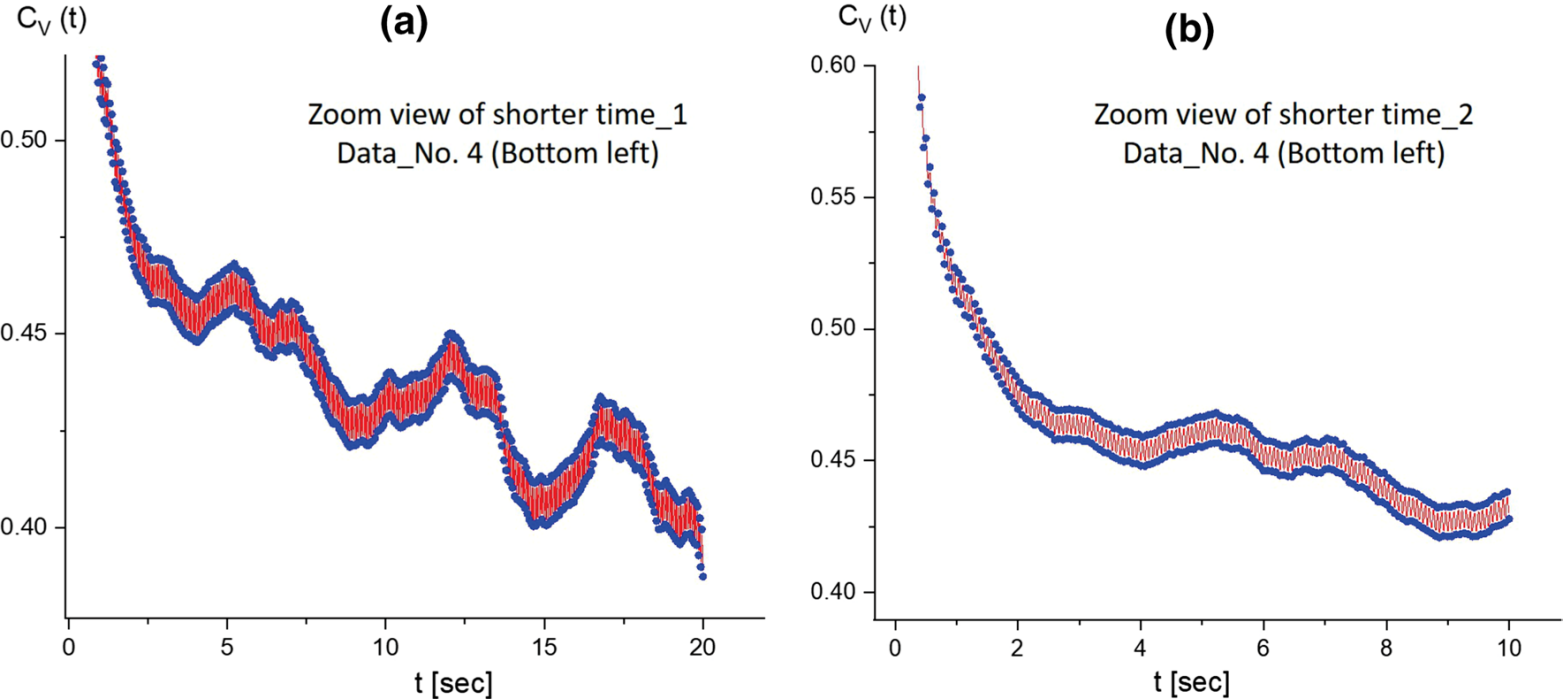
A zoomed-in view of the image–time correlation functions in Fig. 4 for the shorter time spans of **a** 20 s and **b** 10 s. Here, the red regions are filled with the “splitting”, in the zoomed-in view of the correlation function, as the modulating lines between the upper and lower data points. This qualitatively measures the local “motile” fluctuating T4 DNA strand for thermal Brownian motions, which is shown in the amplitude of correlations

To discuss the various conformations of long T4 DNA strands at a low ionic strength of hydrophobic salt (1 mM PPh4Cl), we begin with the bottom-left region of dataset number 4 (Data No. 4 in Fig. 2, available online as Movie 1) (Fig. 4). A stretched single T4 DNA strand is shown and analyzed with the corresponding kinetic morphology of the T4 DNA strand (in Fig. 4, where time-lapsed images are indicated as red pins) in the correlation function. It should be noted that the unique results of the image–time correlation turn out to be a splitting of the data in the zoomed-in view of the correlation function (shown at shorter time-spans of 20 s and 10 s in Fig. 5a and b, respectively). This is a direct consequence of the existing motile motions of long DNA strands. The correlation functions exhibit a relatively high-frequency oscillatory behavior superimposed on the overall slower decay of the correlation function. This kind of oscillatory behavior is not observed for other types of systems using the same equipment, so that the origin of the fast oscillatory behavior cannot be attributed to electronics, sample vibrations or any other source other than the thermal fluctuations of the DNA chain. It is not clear what the origin of these fast oscillations could be: one possibility is that they relate to thermal fluctuations of the local chain length, leading to an elastic oscillatory type of relaxation of the DNA chains. Thus, the correlation function corresponding to the upper and lower envelope of the oscillations are shown on shorter time scales, leading to a “splitting” of the correlation functions. The time resolution is not sufficient to resolve the precise functional form of the oscillations: the time resolution is about 1/30 s, corresponding to a frame rate of 30 fps. Here, the red regions are filled with the modulating lines between upper and lower data points, qualitatively indicating that the amplitude of a locally fluctuating T4 DNA strand is the measure for thermal Brownian motions depicted by the image–time correlation. Similar results can be seen in Fig. 12b for different intensity distributions along the single DNA strands. A greater splitting indicates more motile motions, accompanied by elasticity (see also Figs. 5a and 11a). This agrees with an earlier observation for the Brownian motion of thermal fluctuations for giant DNA strands, of both translational and rotational 2D diffusion, directly observed by a fluorescence microscope (Matsumoto et al. 1992), which confirms that the Rouse’Zimm model is valid for the diluted concentration.

Here, the systematic data analyses of image–time correlation spectroscopy are presented with corresponding morphological changes for various thermal fluctuations of T4 DNA strands, collected by video camera imaging (as shown in Fig. 2). In addition, for a given data movie, different region of interest (ROI) views are selected in a flexible manner for data analysis, as highlighted in the following. The results of image–time correlations are provided for different conformations of long T4 DNA strands, from single- to multiple-interacting strands, including their features. We start with the results of image–time correlations for thermal fluctuations of various conformations with the elasticity of single T4 DNA strands, followed by the results of condensate state T4 DNA globules, with an increase in the ionic strength of the hydrophobic antagonistic salt (PPh4Cl).

**Fig. 6:**
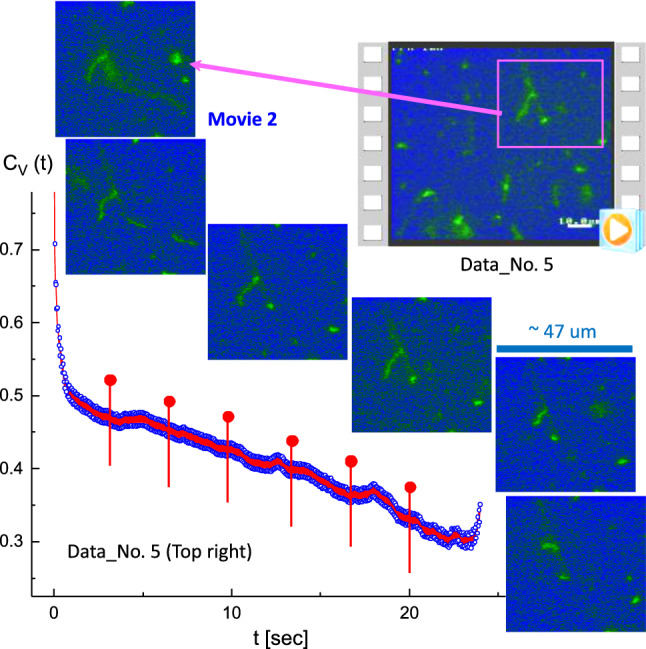
Slightly attached T4 DNA strands are analyzed for disconnection, with the corresponding time-lapsed images indicated as red pins in the correlation function. This is a result of the image–time correlation in the top-right region of Data No. 5 (see Fig. 2; Movie 2 in the supplementary data). The ROI is $300 \times 300$ pixels

**Fig. 7:**
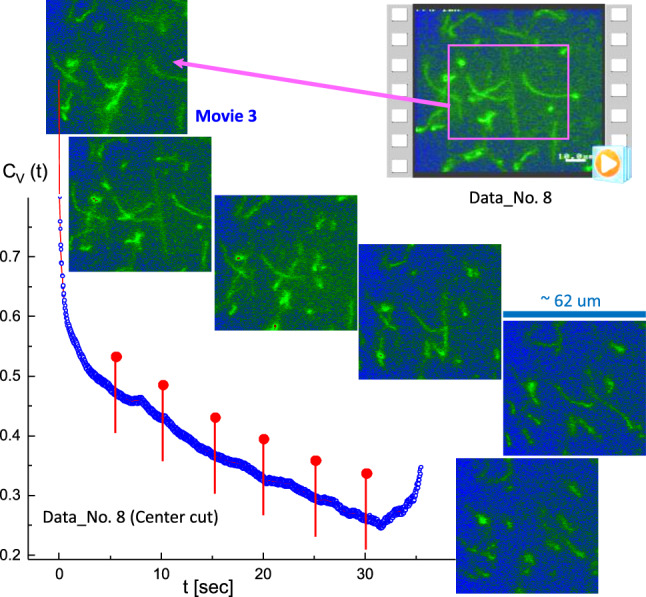
Several attached T4 DNA strands appear to be rearranged in the plane direction, with the corresponding time-lapsed images indicated as red pins in the correlation function. This is a result of the image–time correlation in the central boxed region of Data No. 8 (see Fig. 2; Movie 3 in the supplementary data). The ROI is $394 \times 394$ pixels

**Fig. 8:**
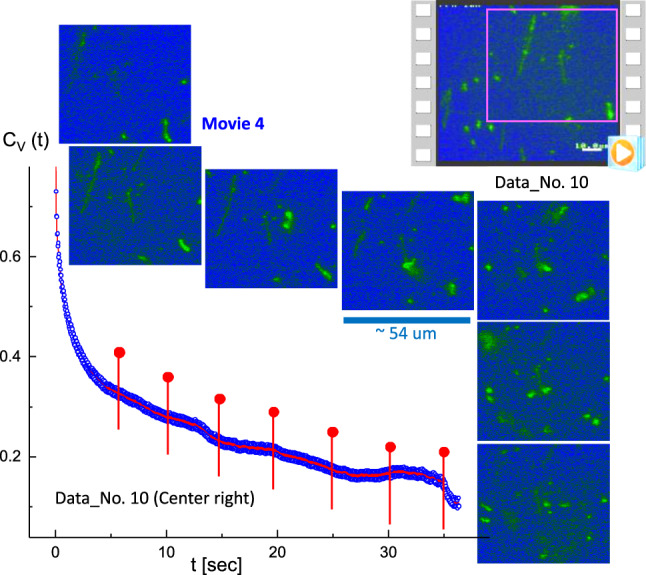
Two or three T4 DNA strands transform to globules, with the corresponding time-lapsed images indicated as red pins in the correlation function. This is the result of the image–time correlation in the center-right region of Data No. 10 (Fig. 2; Movie 4 in the supplementary data). The ROI is $350 \times 350$ pixels

When the long T4 DNA strands were slightly attached (Data No. 5; top-right region in Movie 2), the results of the image–time correlation were analyzed for their disconnection, corresponding to time-lapsed images that are indicated as red pins in the correlation function (in Fig. 6). Several attached T4 DNA strands appear to be rearranged in a plane direction in Data No. 8 (central boxed region in Movie 3), as shown in Fig. 7. Furthermore, two or three T4 DNA strands transform to globules (Fig. 8) in Data No. 10 (center-right region in Movie 4), with corresponding time-lapsed images, and a less pronounced decay seen in the image–time correlation.

**Fig. 9:**
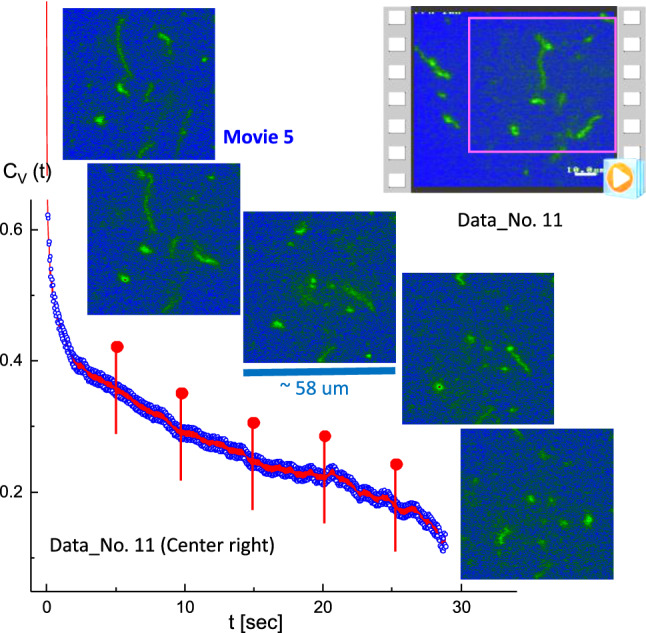
The specifically “folded” T4 DNA strand with several strands in the background, with the corresponding time-lapsed images indicated as red pins in the correlation function. This is the result of the image–time correlation in the center-right region of Data No. 11 (Movie 5 in the supplementary data). The ROI is $370 \times 370$ pixels

**Fig. 10:**
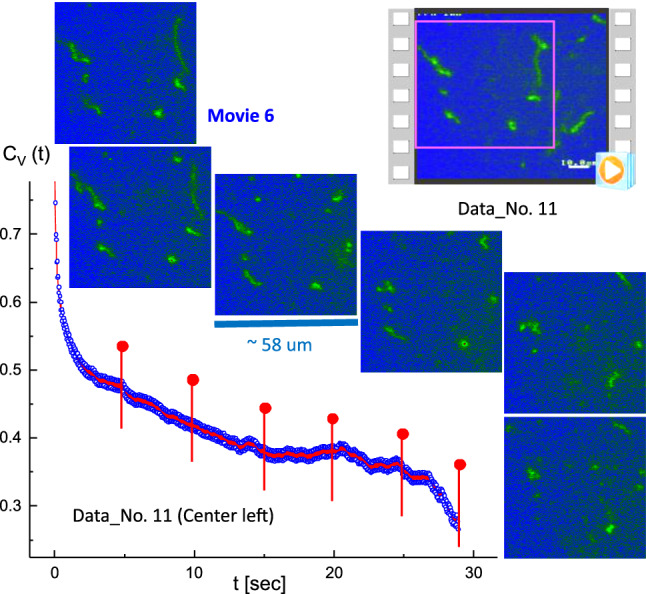
The specifically “folded” T4 DNA strand with several strands in the background, with the corresponding time-lapsed images indicated as red pins in the correlation function. This is the result of the image–time correlation in the center-left region of Data No. 11 (Movie 6 in the supplementary data). The ROI is $370 \times 370$ pixels

**Fig. 11:**
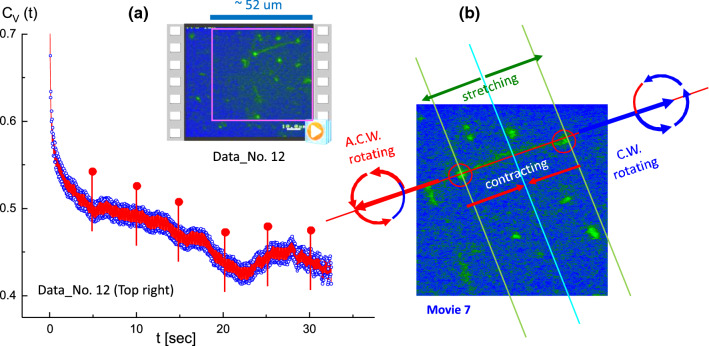
**a** The T4 DNA strand is stretched by two junction points (possibly attached to the bottom of the glass surface). This is the result of the image–time correlation in the top-right region of Data No. 12 (Movie 7 in the supplementary data) with corresponding video data. **b** A basic illustration of the stretched T4 DNA-strands, indicating the asymmetric rotational motions of both end tips. In addition, constant stretching and contracting motions are measured along the stretched directions of the strands, which is seen as a consequence of the large amplitudes in the image–time correlation for its local fluctuations at a time span of 30 s. Corresponding spatio-temporal images are shown in Fig. 13. The ROI is $330 \times 330$ pixels

**Fig. 12:**
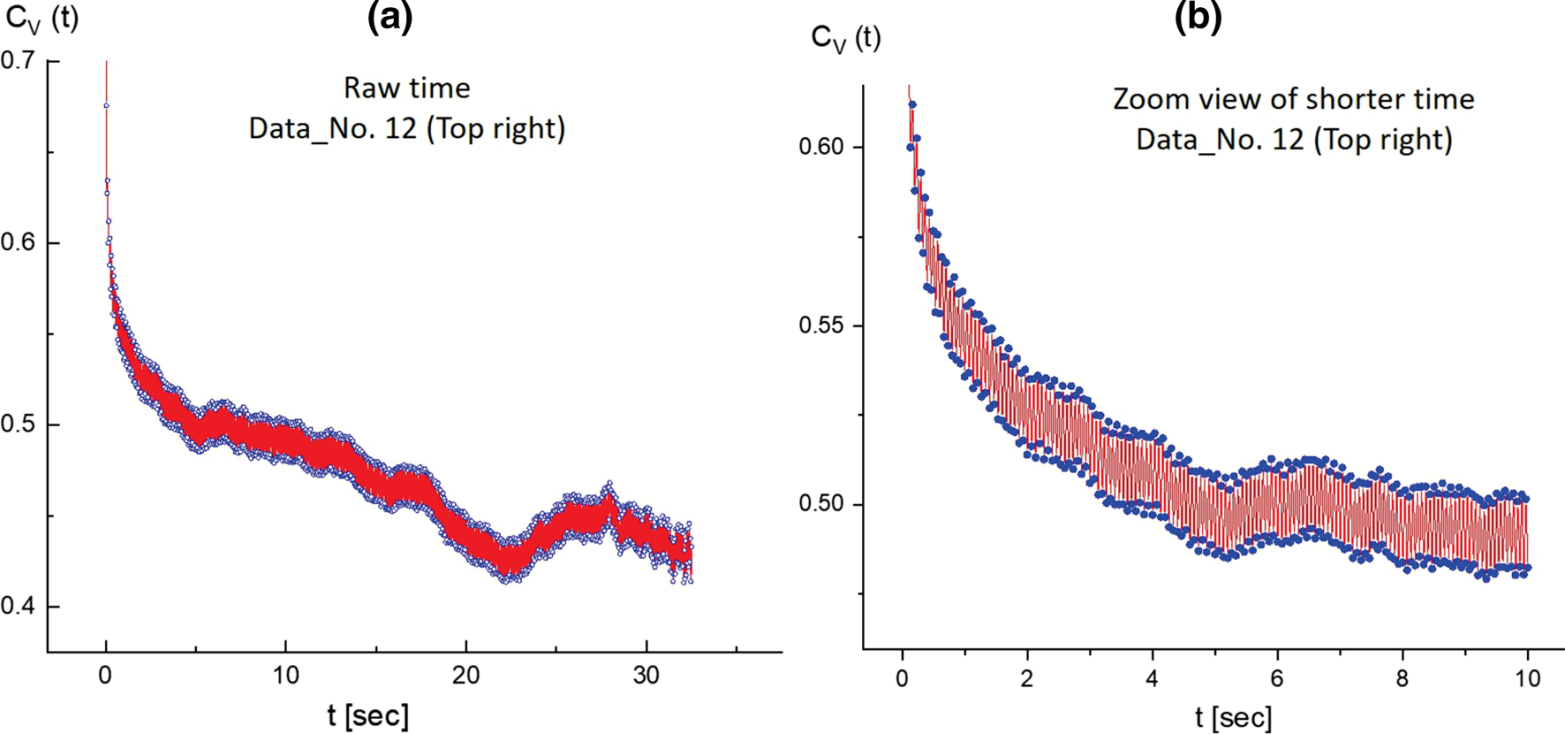
Zoomed-in views of the image–time correlation functions in the top-right region of Data No. 12: **a** at a time span of 30 s, and **b** at a shorter time span of 10 s. Highly pronounced modulations of the local amplitudes are clearly obtained in the image–time correlation function in (**b**) for a particularly tightly stretched T4 DNA strand actively exhibiting motion with two counter-rotating end joints

**Fig. 13:**
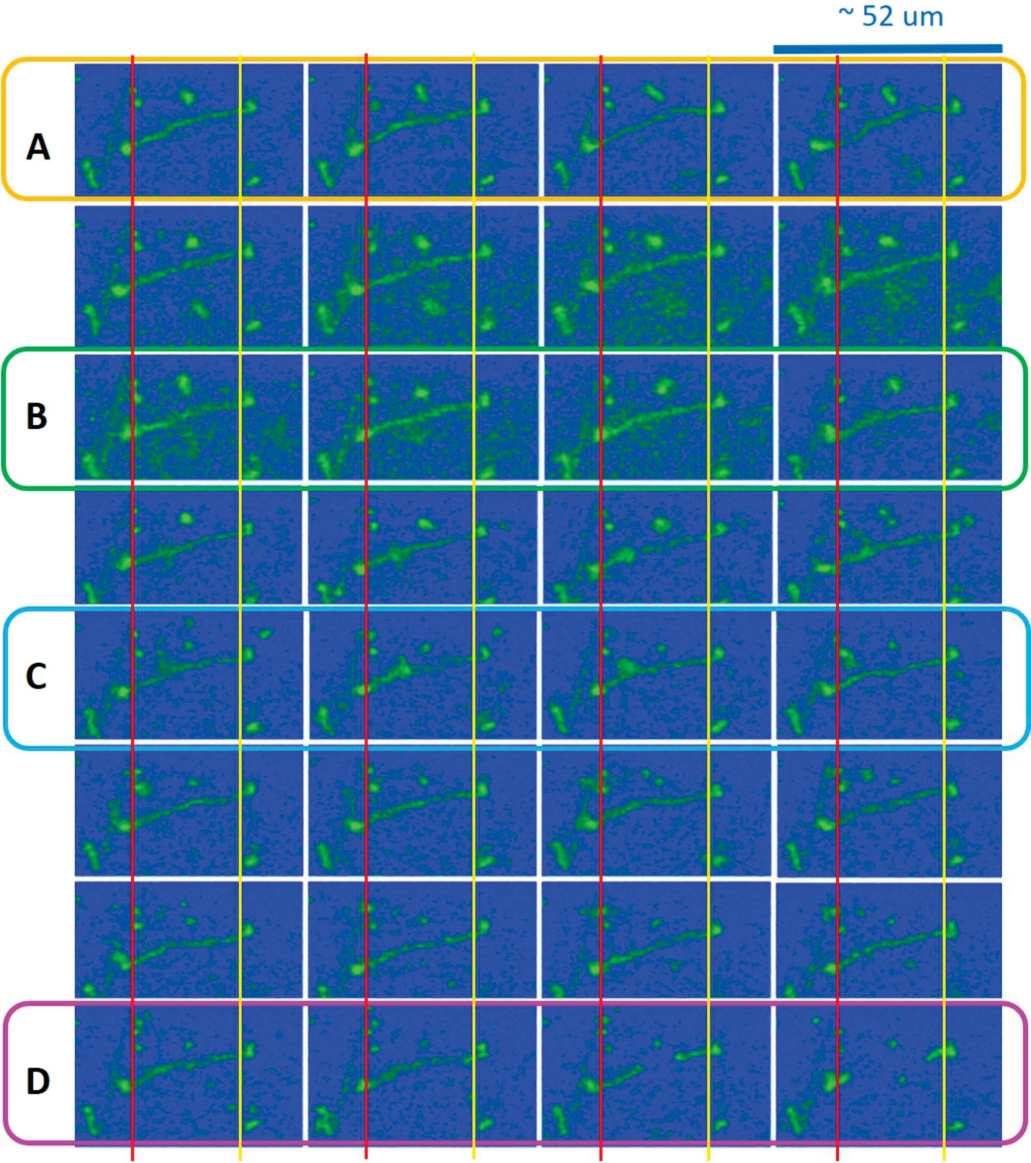
The spatio-temporal images in the top-right region of Data No. 12 (Movie 7) for the connected T4 DNA strand. Different states of A, B, C, and D are shown in time-lapsed conformations as a visual guide, featuring connected and stretched to thin strands (in the A state), contracted to thicker strands (in the B state), locally non-uniform stretched strands (in the C state), and detached strands (in the D state). Here, the time step is about 0.33 s. The vertical lines indicate the translocation of two end points

**Fig. 14:**
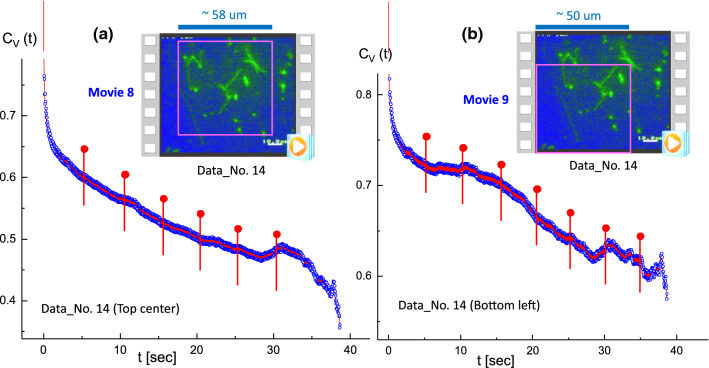
Results of the image–time correlation functions in Data No. 14 for comparison of two different regions: **a** top-center region (in Movie 8), and **b** bottom-left region (in Movie 9). The decay time of an image–time correlation function is directly visualized for the dynamic images, where the edge of morphology (in **b**) decays earlier, but exhibits a plateau due to the existence of a very long T4 DNA strand (see Fig. 16), than the images of shorter lengths for several more entangled strands (in **a**), which is shown in Fig. 15

**Fig. 15:**
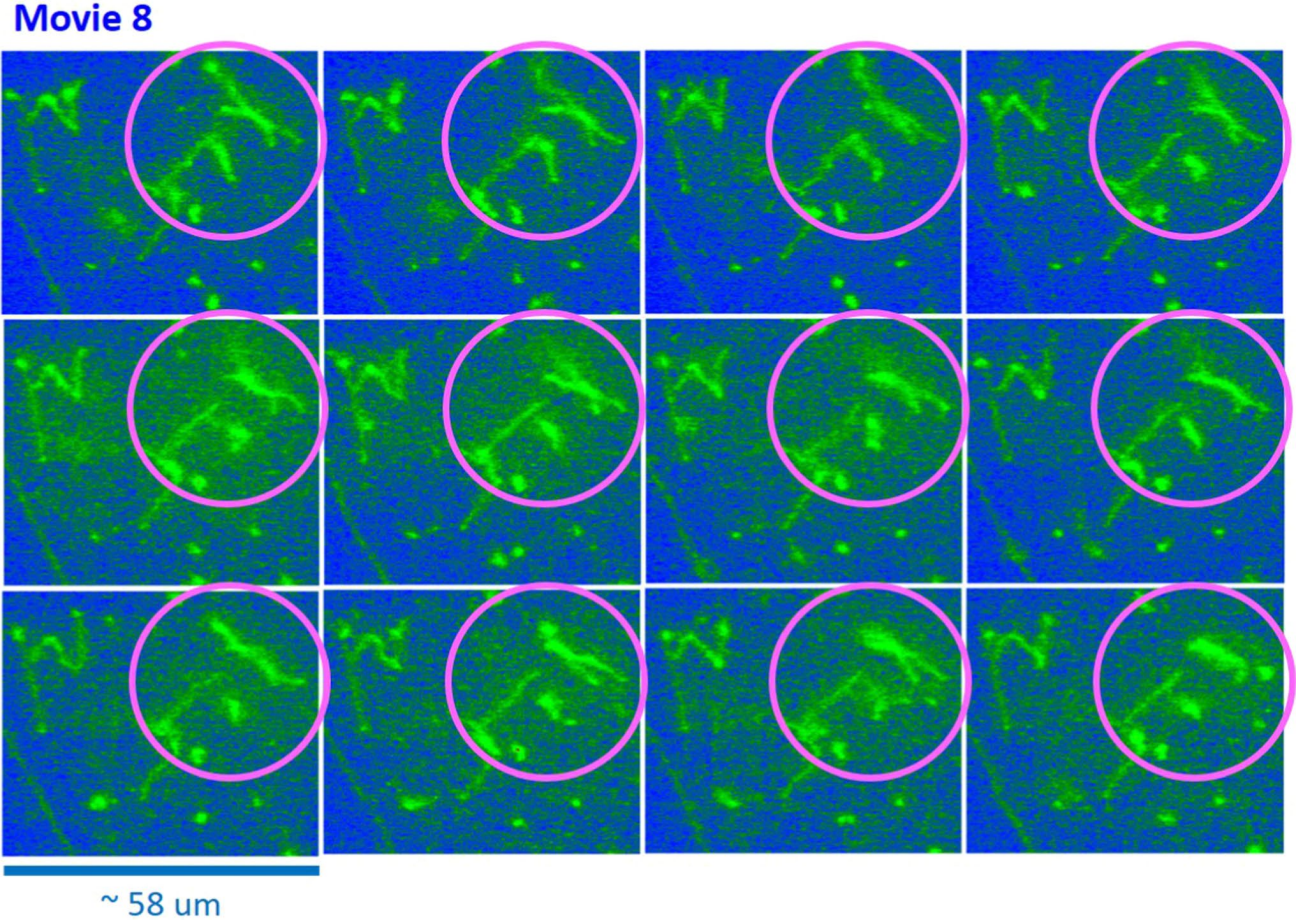
The spatio-temporal images in the top-center region of Data No. 14 (in Movie 8), used in Fig. 15a, for the connected T4 DNA strand. Here, the time step is about 1 s (for 12 s). The particularly significant region of T4 DNA strands is indicated as a pink circle, demonstrating the local variations. The ROI is $370 \times 370$ pixels

**Fig. 16:**
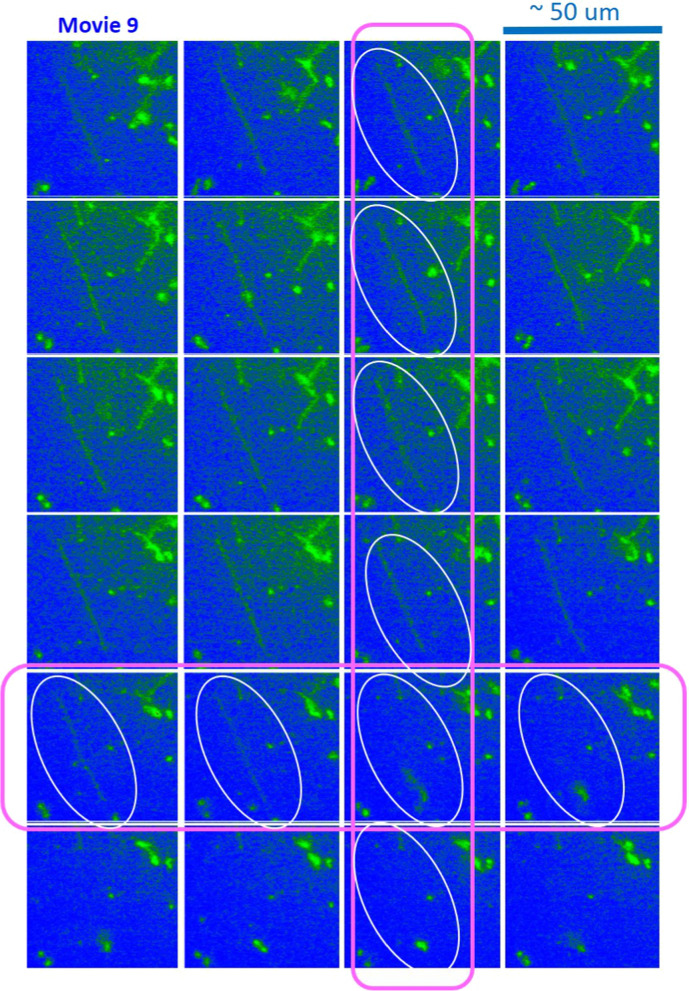
The spatio-temporal images in the bottom-left region in Data No. 14 (in Movie 9), used in Fig. 15b, for the connected T4 DNA strand. Here, the time step is about 1.66 s (for 38.2 s). Of particular interest is the change in the very long stretched DNA strand, becoming disconnected for the given time span, as indicated by the white ovals in the figure. The ROI is $270 \times 270$ pixels. Note that the vertically stretched T4 DNA strand is about $40\;\upmu m$, as indicated in the white ovals

The benefit of an image–time correlation is the flexible degree of freedom in sampling, both in terms of space and time, such that the relevant motions of dynamical images can still be rigorously quantified using the transmitted 2D pixel-pixel intensity correlations and the statistical averaging with variable time binning. Such examples are shown in Figs. 9 and 10 for comparison in the center-right region (Movie 5) and the center-left region (Movie 6), respectively, in Data No. 11. As can be clearly seen, qualitatively different image–time correlation functions are obtained in the selected regions of Data No. 11: (i) the center-right region (in Movie 5) is relevant for a specifically folded T4 DNA strand with several strands in the background (Fig. 9), while the center-left region (in Movie 6) is relevant for the same folded T4 DNA strand along with other strands (Fig. 10). (ii) In Data No. 14, the top-center region (in Movie 8) is relevant for the T4 DNA strand with other interacting strands (Fig. 15), while the bottom-left region (in Movie 9) is for motile T4 DNA strands with several immobile ones in the background (Fig. 16).

### Elasticity of single motile T4 DNA-strands at a low ionic strength of the hydrophobic salt

As seen in Fig. 11, the local elasticity of motile T4 DNA strands is observed in the top-right region of Data No. 12 (Movie 7) as the repetitions of “stretching” and “contracting” motions along the T4 DNA strand (see Fig. 11b). The image–time correlation is shown with a rather pronounced amplitude of the modulation due to an existing visible motion of the stretched (and contracted) DNA strand, which is connected at two junction points. This is caused by DNA strands possibly attaching to the bottom of the treated sample holder surface. A basic illustration of such stretched T4 DNA strands is shown with asymmetric rotational motions of both end tips in Fig. 12b. This can be clearly seen in Movie 7 in the supplementary data.

As a consequence, the most pronounced splitting is shown in the correlation function (in Fig. 11) for the motile motions in the amplitude of the local fluctuating T4 DNA strand as thermal Brownian motions. Zoomed-in views of the image–time correlation functions in Data No. 12 (top-right region) are provided at different time spans of 30 s and 10 s in Fig. 12a and b, respectively. The modulations of local amplitudes are clearly depicted as connected to two counter-rotating end joints in the image–time correlation function (see Fig. 12b) for the particularly tightly stretched T4 DNA strand exhibiting active motile motion. This direct visualization of local motile elasticity in T4 DNA strands is presented in Fig. 13 for Data No. 12 (top-right region). Here, the deviations of vertical red and yellow lines are indicated away from two junction points connected to the DNA strand, as shown in the spatio-temporal images. Dynamic motions of a single T4 strand are highlighted by noting the different states of A, B, C, and D in time-lapsed conformations for less than about 11 s, featuring the connected and stretched to thin strands (A state), contracted to thicker strands (B state), locally non-uniform stretched strands (C state), and, finally, the disconnected T4 DNA strand, separating to two shorter strands (D state).

**Fig. 17:**
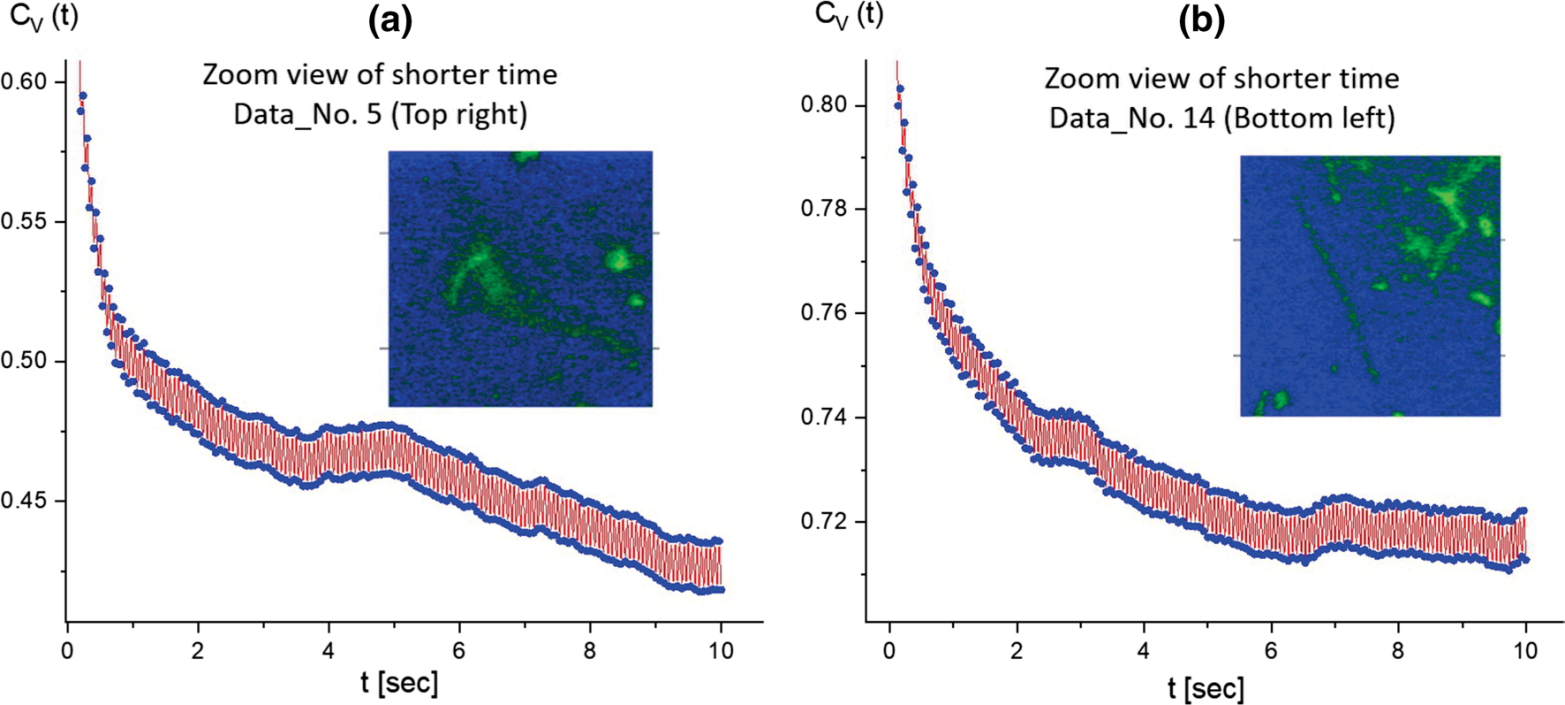
Zoomed-in views of the image–time correlation functions at the shorter time span of 10 s: **a** in the top-right region of Data No. 5, and **b** in the bottom-left region of Data No. 14. Here, the red regions are filled by the modulating lines between upper and lower data points, qualitatively indicating the amplitude of the local fluctuating T4 DNA strand as its own thermal Brownian motions

Other cases are also taken for the local elasticity of T4 DNA strands (in Data No. 14) for the comparison of two different regions: one is relevant for several interacting, multi-contacted DNA strands (top-center region in Movie 8) and the other is a disconnected, fully stretched single DNA strand ($40\;\upmu m$ in length, in the bottom-left region in Movie 9), as shown in Figs. 15, 16, respectively.

**Fig. 18:**
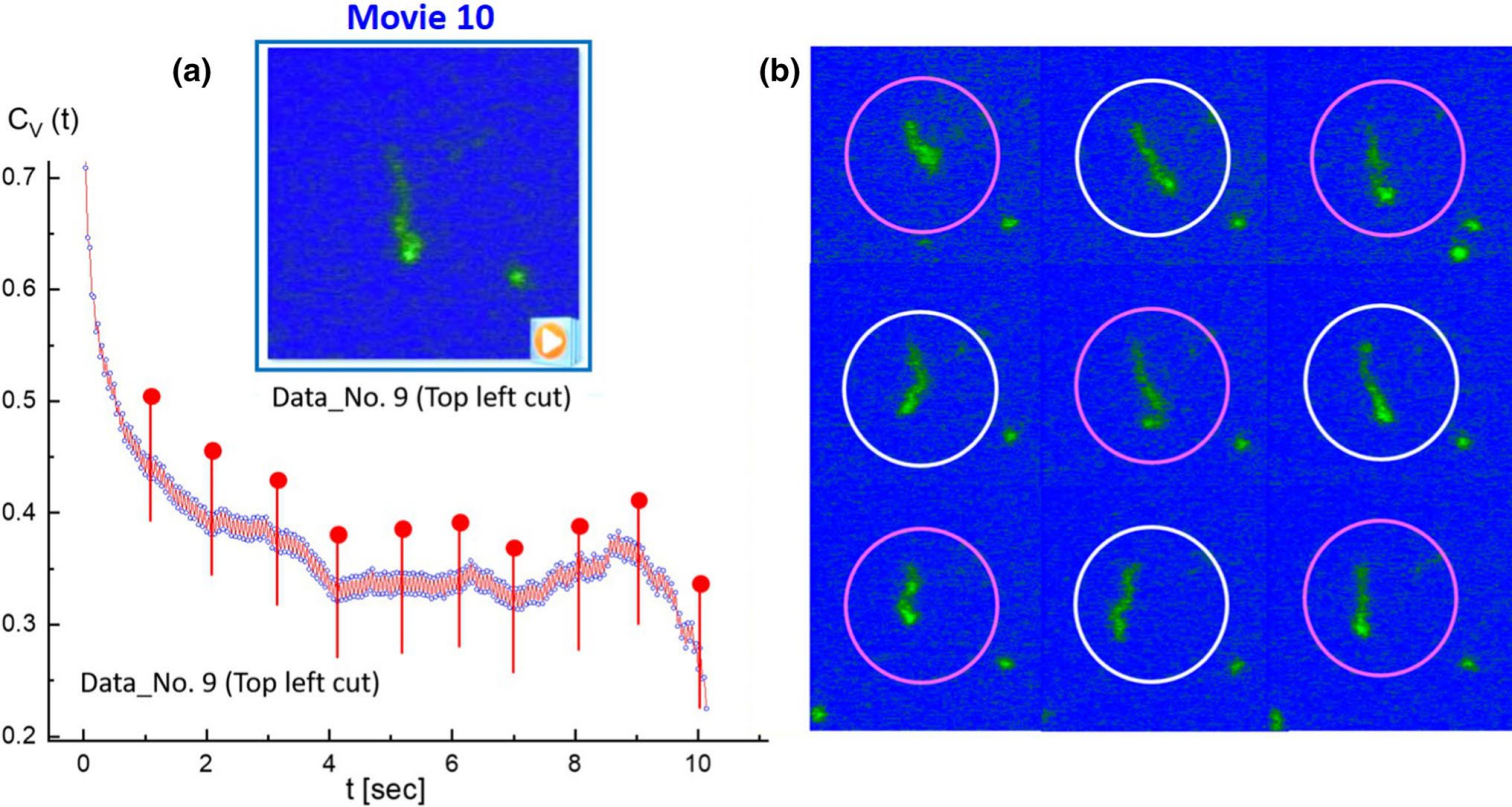
**a** The single fluctuating T4 DNA strand: results of the image–time correlation functions in the top-left cut of Data No. 9 (in Movie 10). The decay time of an image–time correlation function reaches a low saturated value at a time span of less than 5*s*. **b** The variances of local elasticity are shown in the time step of a corresponding conformation in 1 s

The characteristic decays of image–time correlation functions depend on the nature of corresponding dynamics in images, such that the long T4 DNA strand decays earlier to reach a low plateau value (see Fig. 16), while the images of shorter lengths for several more entangled strands decay slower, as shown in Fig. 14a. This can be explained by the fact that T4 DNA strands are now more crowded and partly entangled, as indicated by the pink circles in Fig. 15. A rapid change of the very long stretched DNA strand can be seen in Fig. 14b, becoming disconnected for the given time span of 10 s, indicated by the white ovals in the vertical arrangement in Fig. 16.

To validate further the accuracy of an apparent splitting in the image–time correlation spectroscopy magnitude, two sets of data movies were obtained independently at a short time span of 10 s: (i) one for the image–time correlation of several interacting T4 DNA strands, comparing statistics for a fewer number of DNA strands (Data No. 5, top-right region), and a larger number of DNA strands (for Data No. 14, bottom-left). Similar fluctuating amplitudes are shown in the local modulations (see the red lines in Fig. 17a and b) for thermal Brownian motions of averaged ensembles, irrespective of different samplings for interacting long T4 DNA strands. (ii) The other is an image–time correlation for a single fluctuating motile T4 DNA strand, in Fig. 18a, from the top-left region in Data No. 9 (see Movie 10), the local conformation changes can be clearly seen (in Fig. 18b) in the time step of 1 s, where the initial decay is shown to be about 5 s with the variances in local elasticity originating from the non-uniform motility of the T4 DNA strand itself.

**Fig. 19:**
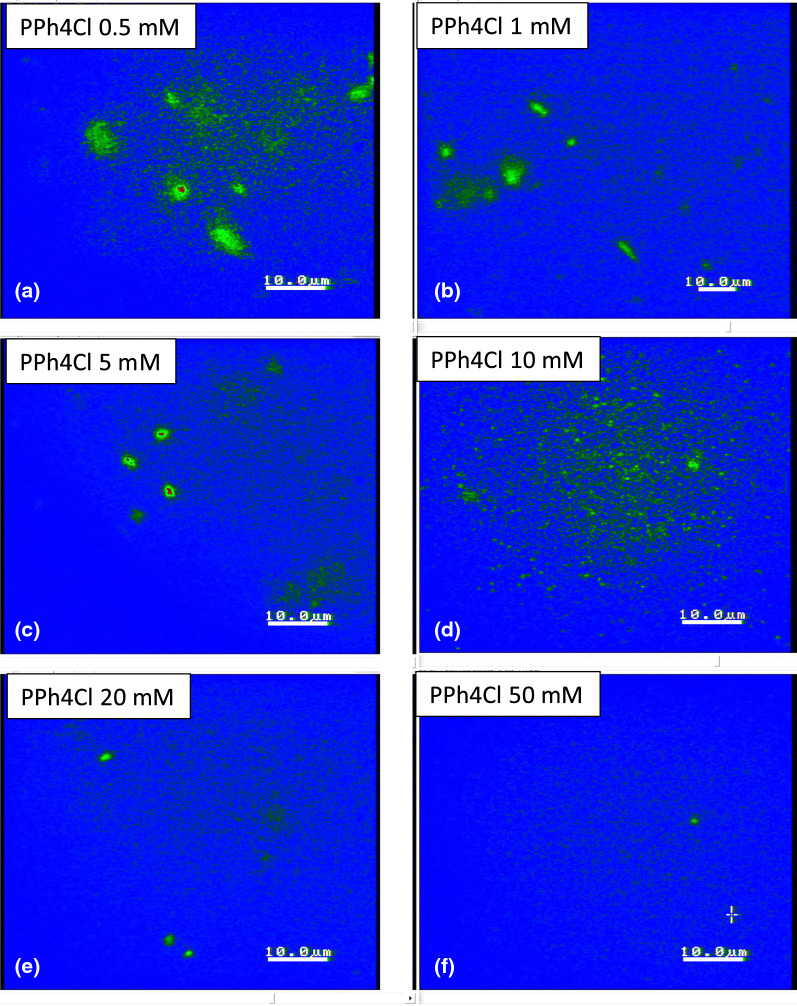
The effect of the ionic strength of hydrophobic antagonistic salt (PPh4Cl) on the bacteriophage T4 DNA:**a** 0.5 mM, **b** 1 mM, **c** 5 mM, **d** 10 mM, **e** 20 mM, and **f** 50 mM. As the ionic strength increases, fewer DNA strands are visible against the background of a salt solution. This might be due to the strong electrostatic screening or the precipitation of highly non-uniformly charged DNA condensate aggregates

### Electrostatic interactions of T4 DNA via the effect of hydrophobic antagonistic salt

**Fig. 20:**
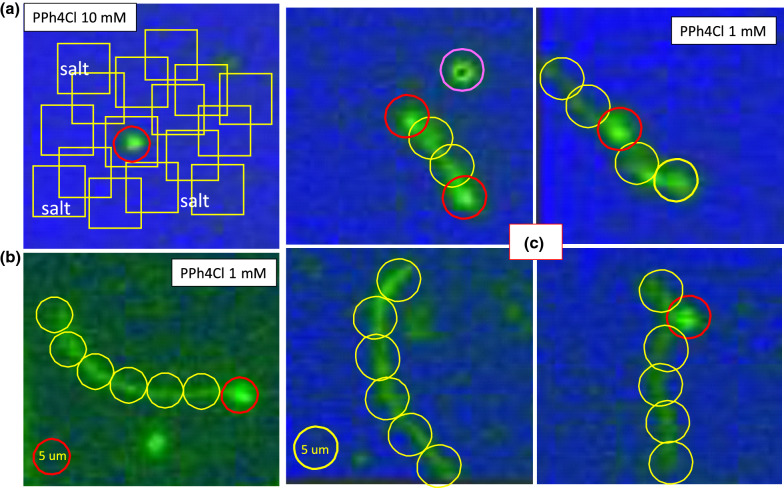
Potential mechanisms of driving conformation changes of T4 DNA at high and low ionic strengths of hydrophobic–cationic antagonistic PPh4Cl salts: **a** at 10 mM, and **b**, **c** at 1 mM. Condensate T4 DNA globules are clearly observed due to the short-ranged attractive interactions of T4 and PPh4Cl as strongly screened in the electrostatic interactions. In contrast, at a low ionic strength, rather pronounced thermal fluctuations of Brownian motions for single, multiple, and even entangled T4 DNA strands can be seen. A few examples of such robust thermal fluctuations of T4 DNA strands are clearly illustrated between more motile (red circle) and immobile strands (yellow circles) in **c**. For further information, see Movie 5 in the Supplementary data

A possible non-uniform local elasticity (including motility) can be briefly addressed as follows: By increasing the ionic strength of the monovalent hydrophobic antagonistic salt (PPh4Cl) solution, long T4 DNA strands become the condensates of globules, as illustrated earlier in Fig. 1 (upper panel). Our actual measurements concerning the effect of a hydrophobic antagonistic salt on the bacteriophage T4 DNA are shown in Fig. 19 by varying the following ionic strengths: 0.5 mM, 1 mM, 5 mM, 10 mM, 20 mM, and 50 mM. It is worth noting that fewer T4 DNAs (in the green region) are obtained in combination with the salt solution (in the blue background). This also confirms that the electrostatic interaction plays a role in the screening effect, which might be relevant to the formation of a strongly correlated liquid (SCL) of counterions at the DNA surface. Thus, charge inversion by multivalent ions is made possible through the electro-photoelectric mobility of condensed DNA, which is detected by magnetic tweezers (Besteman et al. 2007). With an ionic strength of 1–10 mM, the transformation of a DNA strand to a condensed DNA globule occurs under the same applied force (2–3 pN) with relatively broad distributions, while for the higher ionic strength of 50 mM, the force is measured as a narrower distribution. This supports the notion that the condensed DNA globules are more accessible at a higher ionic strength, which also agrees with our results (in Fig. 19) and that fewer T4 DNA strands are observed against the background of a salt solution. In this respect, the strong electrostatic screening plays a primary role, as opposed to the precipitations of highly non-uniformly charged DNA condensate aggregates. However, the latter case cannot be ruled out in terms of microscopic dynamics dependent on ionic strength. A simple explanation of a driving mechanism for the fluctuation of T4 DNA, in reaction to hydrophobic antagonistic salts at a high and low ionic strength, is depicted in Fig. 20a and b, respectively. At a higher ionic strength (above 10 mM) of the hydrophobic salt, strong electrostatic screening might take place, as shown in Fig. 20a for the square lattice in the solvent background. The condensed T4 DNA globules are observed due to the short-ranged attractive interactions of T4 DNA and PPh4Cl salt at a high ionic strength, which are screened strongly by the cutoff in electrostatic interactions. In contrast, at a low ionic strength (of 1 mM), clearly visible T4 DNA strands are frequently seen in Fig. 20b, possibly due to the condensed ions and diffusive mobile ions surrounding the T4 DNA. Pronounced thermal fluctuations of Brownian motions are therefore valid for the single, multiple, and even entangled T4 DNA strands. In the case of robust thermal fluctuations for the motile T4 DNA strand, even the active motile sites (red circle) can be localized as leading DNA conformation changes, shown in simplified features of Fig. 20c, distinguished from the immobile sites (yellow circles). The overlapping then appears to occur more frequently by motile sites that are also connected to an immobile site. This might indicate that the interaction parameter of the second viral coefficient becomes important for multiple DNA strands at large corresponding aspect ratios, even for a low concentration (Hill 1960).

## Conclusion

In this paper, we have shown the mesoscopic time scale thermal fluctuations of bacteriophage T4 long DNAs, including their intrinsic motility and local elasticity, analyzed by image–time correlation spectroscopy. It has shown clearly not only the useful validity of an image–time correlation for analyzing large biomacromolecules, but also provided insights into effects of hydrophobic antagonistic salt on active bacteriophage T4 long DNA strands, including thermal translocations in electrostatic interactions. The conformational changes of single and multiple T4 DNA strands were analyzed at a low ionic strength of the cationic antagonistic (PPh4Cl) salt (1 mM). A surprising result was found for the motility of the long DNA strand, revealed by the splitting of modulations in the correlations, featuring real-time local fluctuations of existing non-uniform elasticity for various DNA strand conformations. By increasing the ionic strength, less condensates of T4 DNA globules were formed due to strong electrostatic screening (above 5–10 mM hydrophobic salt). The possible explanation for the hydrophobic–cationic salt-induced elasticity of single T4 DNA strands originates from the strongly correlated liquid (SCL) for counterions on the DNA surface.

In addition, the accuracy of image–time correlation functions maintains reliable for larger fields of regions in the data movies, depicting the average elastic forces acting on the T4 DNA strands in time. There are a number of correlation functions shown that refer to the same video images, but we calculated them from different partial regions from the total field-of-view, to establish the reproducibility of the correlation functions. These correlation functions are similar to within about $5\%$. Correlation functions taken for different systems show qualitative differences. For example, the correlation function for the initially fully unfolded DNA chains in bulk solution in Fig. 4, shows an intermediate slowly relaxing mode, followed by a faster decaying mode that can be ascribed to the ongoing transition from the unfolded to the folded/collapsed state. In addition, within the time window related to the slow relaxing mode, the correlation function shows intermittent increasing values (as can be seen from Fig. 5a), which can be interpreted as the elastic retraction of the chains after thermal excitation leading to chain distortion. The correlation functions related to (partially) collapsed chains exhibit a more pronounced initial decay followed by a slower decay that is, however, faster as compared to the fully unfolded chains. Furthermore, correlation functions are shown, where, for example, a mixture of unfolded and folded/collapsed chains are present. For these mixed systems, the decay rate after the fast-initial decay is in between those for the pure systems. There is, however, no clear two-mode decay observed (after the fast-initial decay), which can be attributed to the faster Brownian center-of-mass displacements of the small collapsed DNA chains as compared to the unfolded chains, while the relaxation rate corresponding to the chain conformation dynamics is less than an order of magnitude different as compared to the Brownian displacements of the folded/collapsed chains. Thus, we can conclude that local conformational changes of these long T4 DNA strands are depicted accurately by the image–time correlation plots.

The results convincingly show that the correlation function serves as a useful means of quantifying the effect of fluctuating long DNA strands, exploiting their elastic deformations, directly from live images of single- and multi-interacting strands in solvent. We have found that the non-uniform local elasticity of the charged long DNA strand may be related to an active motile site that more frequently overlaps towards immobile ones, resulting in the release of attached hydrophobic salt ions (see Fig. 20c). A similar observation has been made for the coat proteins that assemble on its DNA to form a complete fd bacteriophage, extracted from E. coli bacteria (Marvin and Hohn 1969; Marvin et al. 1994), where the dissociation of condensed ions is responsible for the binding of DNA strands (Kang 2021; Zimmermann et al. 1986). To conclude, the system of hydrophobic-salt-induced reversible DNA strand fluctuations is able both experimentally and theoretically to provide an understand of their active motions. We hope these findings provide useful insights, from the application of image–time correlation, into an understanding of parameters describing interactions of hydrophobic antagonistic salts with long DNA strands. This work contributes to knowledge of the use of image–time correlation for quantifying local activities of large biomolecules.

## Supplementary Information

Below is the link to the electronic supplementary material.

Supplementary material 1 (pdf 53 KB)

Supplementary material 2 (mov 6801 KB)

Supplementary material 3 (mov 5562 KB)

Supplementary material 4 (mov 8579 KB)

Supplementary material 5 (mov 8960 KB)

Supplementary material 6 (mov 7191 KB)

Supplementary material 7 (mov 7168 KB)

Supplementary material 8 (mov 8118 KB)

Supplementary material 9 (mov 9343 KB)

Supplementary material 10 (mov 9424 KB)

Supplementary material 11 (mov 3908 KB)
