# Supplementary material for: Direct visualization of local activities of long DNA strands via image–time correlation
Source: Eur Biophys J. 2021 Sep 9;50(8):1139–55. doi: 10.1007/s00249-021-01570-0 (PMC8566448; doi:10.1007/s00249-021-01570-0)
Supplement: Supplementary file 1 — Supplementary material 1 (pdf 53 KB) [file 249_2021_1570_MOESM1_ESM.pdf]

Supplementary data Movies:

Title: Direct Image-time Correlations of Long DNA Strands: The Effect of Hydrophobic Antagonistic Salt

Authors: Kyongok Kang, Yue Ma, and Koichiro Sadakane

The manuscript contains **10 movies in total**, which play in Windows Media Player and VLC media player:

Movie 1: The region of bottom-left view in Data-4 movie, where a stretched single T4 DNA-strand is interested, with ROI as 250 x 250 pixels, in Fig. 4.

Movie 2: The region of top-right view in Data-5 movie, where slightly attached T4 DNA-strands are interested for the disconnection. ROI is 300 x 300 pixels, in Fig. 6.

Movie 3: The region of center-cut view in Data-8 movie, where few attached T4 DNA-strands seem to be rearranged in a direction (in the plane). ROI is 394 x 394 pixels, in Fig. 7.

Movie 4: The region of center-right view in Data-10 movie, where two or three T4 DNA-strands transit to globules. ROI is 350 x 350 pixels, in Fig. 8.

Movie 5: The region of center-right view in Data-11 movie, where the specifically "folded" T4 DNA strand with few ones in background. ROI is 370 x 370 pixels, in Fig. 9.

Movie 6: The region of center-left view in Data-11 movie, where the specifically "folded" T4 DNA strand with few other ones in background. ROI is 370 x 370 pixels, in Fig. 10.

Movie 7: The region of top-right view in Data-12 movie, where the T4 DNA strand is stretched by two junction points (possibly attached to the bottom of glass surfaces). ROI is 330 x 330 pixels, in Fig. 11.

Movie 8: The top-center view in Data-14 movie, and ROI is 370 x 370 pixels. Their spatiotemporal images are shown in Fig. 14 and in Fig. 15.

Movie 9: The bottom-left view in Data-14 movie, and ROI is 370 x 370 pixels. Their spatiotemporal images are shown in Fig. 14 and Fig. 16.

Movie 10: The top-left cut view in Data-9 movie, for capturing the single fluctuating T4 DNA strand, in Fig. 18.
